# Burden and unmet needs in asthma care in the Asia‐Pacific region

**DOI:** 10.1002/puh2.15

**Published:** 2022-08-29

**Authors:** Surbhi Wadhawan, Umadevi A. Muthukumaru, Li Cher Loh

**Affiliations:** ^1^ Medical Affairs Department Novartis Corporation (Malaysia) Sdn Bhd Petaling Jaya Selangor Malaysia; ^2^ Department of Respiratory Medicine Hospital Pulau Pinang George Town Penang Malaysia; ^3^ Department of Medicine RCSI & UCD Malaysia Campus George Town Penang Malaysia

**Keywords:** burden of disease, chronic respiratory disease, public health, uncontrolled asthma

## Abstract

Asthma is a chronic respiratory disease with increasing global prevalence. It affects approximately 300 million people globally and more than a third of these patients have uncontrolled asthma. The burden of poorly controlled asthma is considerable both for the individuals and the society. The World Health Organization (WHO) estimates that, 455,000 people died from asthma worldwide in 2019, the majority in low and low‐middle‐income countries. This article highlights the unmet needs and burden of asthma in the Asia‐Pacific region where many patients have poorly controlled asthma despite advances in asthma care and treatment. A mismatch between patients’ (and physicians’) perception of asthma control and optimal asthma control as defined by the Global Initiative for Asthma (GINA) is a challenge in the real‐life management of many patients. Consequently, treatment adherence and optimization are affected. In many of these aspects, patients with asthma in the Asia‐Pacific region are comparatively worse than those from Europe and North America. In future, cooperation of all stakeholders is needed in improving awareness, education, and goal‐directed management of asthma with meaningful improvement in quality of life. This article draws attention to some of the important gaps in asthma management in the Asia‐Pacific region and suggests some measures to address these unmet needs.

## INTRODUCTION

Asthma is a chronic disease requiring comprehensive treatment with an aim to reduce the symptom burden (i.e., good symptom control while maintaining normal activity levels) and minimizing the risk of adverse events such as exacerbations, fixed airflow limitation, and treatment side effects [1]. Asthma is characterized by chronic airway inflammation involving both large‐ and small‐conducting airways including structural remodeling in some cases [2]. Asthma pathophysiology is heterogeneous and primarily has two phenotypes based on the presence of airway eosinophilia and T‐cell response: type 2 (allergic asthma) and non‐type 2 (non‐allergic asthma) [2, 3]. Type‐2 asthma, which occurs in majority of children (>80%) and adults with asthma, is characterized by the presence of T2‐high biomarkers including elevated airway and peripheral eosinophils, elevated fractional exhaled nitric oxide (FeNO), and presence of allergen‐specific IgE [2, 3]. On the contrary, the pathophysiology of non‐type‐2 or T2‐low asthma is not well established. It is generally typified by neutrophilic (sputum neutrophils > 40%–60%) or paucigranulocytic (i.e., normal sputum levels of both neutrophils and eosinophils) inflammation and is unresponsive to corticosteroid therapy [2, 3].

Asthma affects approximately 300 million people worldwide and the estimated prevalence is increasing annually and yet 20%–70% remain undiagnosed [4, 5, 6, 7, 8]. People with asthma can lead a normal life if timely and adequate intervention is available. Although, asthma treatment landscape has come a long way from the short‐acting beta‐agonist (SABA) epidemic of the 1960s, asthma still contributes to significant economic burden on the healthcare resources [5, 9, 10, 11]. In the latest Global Asthma Report, asthma ranked 16th among the leading causes of years lived with disability (YLD) and 28th among the leading causes of burden of disease, as measured by disability adjusted life years (DALYs) [5]. Asthma is among the top 20 causes of DALYs for children up to 9 years of age [6]. According to the Global Asthma Report 2018, asthma medications were the highest contributors to the direct medical costs of asthma management in North America and Europe whereas emergency room (ER) visits, physician consultations and outpatient costs contributed largely to the total costs of asthma care in the Middle East and Southeast Asia region [5]. Hence, this article aims to provide an overview of the burden of asthma and the unmet needs in asthma care in the Asia‐Pacific region.

## METHODS

The literature search was performed using two electronic databases, PubMed and ScienceDirect between May and September 2021. The search terms used were 'asthma burden, uncontrolled asthma, study, survey, Asia‐Pacific, and Malaysia'. Relevant asthma studies, surveys, and randomized controlled trials for patients with asthma, published in the last two decades (2000–2021) were included. An emphasis was laid on studies from the Asia‐Pacific region including Malaysia, Singapore, the Philippines, India, Australia, Sri Lanka, China, Hong Kong, Taiwan, Korea, Thailand, Vietnam, and Indonesia. Some of the pivotal asthma studies from Europe were also included to draw comparisons on the asthma burden and unmet needs between Asia‐Pacific and Europe. Single‐center studies, with a small sample size (*n* ≤ 100) or solely on occupational asthma, exercise‐induced asthma, aspirin exacerbated respiratory disease (AERD), asthma and COPD (chronic obstructive pulmonary disease) overlap syndrome, studies including patients with asthma primarily from the United States, South America, and Asian countries other than Asia‐Pacific were excluded. Manuscripts in languages other than English were also excluded.

## GLOBAL BURDEN OF UNCONTROLLED ASTHMA

Global Initiative for Asthma (GINA) recommends symptom control and minimizing the future risk of exacerbations to achieve good asthma control [1]. Despite treatment advances, asthma still contributes considerably to the morbidity and mortality burden [4, 5, 8]. According to the World Health Organization (WHO) estimates, 455,000 people died from asthma worldwide in 2019, the majority in low and low‐middle‐income countries [12]. In 2016, 24% of the patients participating in Asthma UK Annual Asthma Survey reported that asthma symptoms impacted their ability to work or study, with more than a quarter reporting at least 1 week off from work or school because of their asthma in the previous year [13, 14]. It is estimated that up to 65% of the patients with asthma (i.e., around 3.5 million people) had not received optimum asthma care in the United Kingdom (UK) in 2020 as against 61% (i.e., around 3.3 million people) in the previous year [15, 16]. The direct annual cost of asthma care has been reported to be around €17.1 billion, and it is estimated that 10% of the European population suffers from it [17]. Moreover, deaths due to asthma are still being reported [4, 17]. A summary of studies reporting the burden of uncontrolled asthma is shown in Table 1.

**TABLE 1:** Summary of key studies in Europe and Asia‐Pacific region on patients with asthma and physicians treating asthma

**Study**	**Publication year**	**Participating countries**	**Inclusion criteria**	**Study design**	**Sample size (*n*)**	**Key findings**
The Asthma Insights and Reality in Asia‐Pacific (AIRIAP) study [20]	2002	Urban areas from mainland China, Hong Kong, Korea, Malaysia, the Philippines, Singapore, Taiwan, and Vietnam	Adults and parents or caregivers of children with asthma	Multicenter, cross‐sectional, observational	3207	Uncontrolled in 51.4% of the respondents. Controller asthma medication (primarily ICS) used in only 14% of the respondents. One in three with perceived well‐controlled asthma had severe persistent asthma [20].
Asthma Insights and Reality in Asia‐Pacific Phase 2 (AIRIAP 2) (AIRIAP Phase 2) [21]	2011	Urban areas from mainland China, Hong Kong, Korea, Malaysia, the Philippines, Singapore, Taiwan, Vietnam, Sri Lanka, Thailand, India, and Indonesia	Adults and parents or caregivers of children with asthma	Community based, cross‐sectional, observational	4805	More than half (56%) had uncontrolled asthma based on ACT scores and had significantly higher healthcare utilization in the form of ER visits and hospitalization [21].
Asia‐Pacific Asthma Insights and Management (AP‐AIM) [23]	2014	Australia, China, Hong Kong, India, Malaysia, Singapore, South Korea, Taiwan, and Thailand	Patients (12 years and older) with confirmed diagnosis of asthma and taking asthma medication or had an exacerbation in the past 12 months	Cross‐sectional, observational	3630	Partly‐controlled and uncontrolled asthma cohorts had more healthcare resource utilization and reported greater impact of asthma on their QoL as reflected in missed school/ work days and social as well as psychological wellbeing [23].
REcognise Asthma and LInk to Symptoms and Experience (REALISE) [29]	2014	Austria, Belgium, Finland, France, Germany, Italy, the Netherlands, Norway, Spain, Sweden and the United Kingdom (UK)	Individuals with asthma (18 ‐ 50 years old; ≥2 prescriptions in the previous two years; used of social media)	Cross‐sectional, observational	8000	Majority (80%) partly‐controlled or uncontrolled. Around 40% reported higher than recommended use (three or more times in the previous week) of RI. HTN and depression most frequently self‐reported comorbidities with a prevalence of 15% and 13.5% respectively [29].
The REcognise Asthma and LInk to Symptoms and Experience Asia study (REALISE‐ASIA) [30]	2015	Korea, Hong Kong, Indonesia, Malaysia, People's Republic of China, Philippines, Singapore, and Taiwan	Individuals with asthma (18 ‐ 50 years old; ≥2 prescriptions in the previous two years; used of social media)	Cross‐sectional, observational	2467	Majority (80%) partly‐controlled or uncontrolled. QoL impacted and reflected in missed workdays, ER visits (38.4%) and hospitalization (33.1%). Asthma control perceived as management of exacerbations instead of prevention [30].
Global Asthma Physician Survey (GAPS) [40]	2017	Australia, Canada, China, France, Germany, and Japan	General Practitioners and Internal Medicine physicians routinely seeing patients with asthma (≥4 in a month)	Multicenter, observational, cross‐sectional survey	1809	Physician perceived barriers to patients’ treatment adherence included as needed use of medication, symptom tolerance and lack of understanding of treatment benefits. Written AAP provided to 37% patients. Overall, 10% physicians used validated asthma questionnaires to assess control [40].
Still Fighting for Breath [31]	2018	Brazil, Canada, France, Germany, Italy, Japan, Portugal, Spain, and UK	Individuals with severe persistent asthma and included both adults (aged > 18 years) and parents or caregivers for children (6–17 years old)	Observational, cross‐sectional	1333	Similar to Fighting for Breath survey (2004‐05). Discrepancy between patient perceived asthma control (42%) vs. the GINA defined control (6%). Greater prevalence (5 ‐ 12 fold) of anxiety in patients. Up to 88% had daily living disruptions and 97% had sleep disruptions [31].
Subjective Evaluation of Asthma CONtrol (SEACON) [39]	2019	Japan	Patients treated for asthma with at least 4 reviews in the past 1 year and no exacerbation episode in preceding 1 month of the study were recruited	Observational, multicenter, cross‐sectional study	1697	Discordance between physicians’ perception and patients’ asthma control as assessed by ACQ‐5 scores. Discordance rates higher for patients whose asthma was not well controlled and patients who had not received spirometry assessment [39].
Asthma Control Level in Primary Care Setting in Malaysia (ASCOPE) [28]	2020	Malaysia	18 years and older; outpatients	Prospective, observational, multicenter study at primary healthcare centers	1011	Six in ten patients not optimally controlled. Treatment adherence poor in almost half of the patients. Frequent OCS (5 days or more course within 3 months) and higher SABA usage (> 1×200‐dose canister/month) observed primarily in partly‐controlled and uncontrolled cohort [28].
United Kingdom Clinical Practice Research Datalink (CPRD) study [24]	2020	The United Kingdom	Data collected from a network of general physicians (GP) practices across the UK (2006‐2016) through anonymized longitudinal medical records (CPRD). The patient population in CPRD was representative of the UK primary care population	Retrospective, observational, cohort study	Overall, 45,804 patients with asthma (29,229 and 16,575 patients initiated medium‐ and high‐dose ICS‐LABA respectively) were monitored	Around 35% and 46% patients on MD and HD ICS/LABA respectively were uncontrolled. Less than half were adherent to the treatment. Seven out of ten compliant patients on HD ICS found to be uncontrolled. Comparatively higher healthcare resource utilization in uncontrolled cohort [24].

Abbreviations: AAP, Asthma Action Plan; ACQ‐5, Asthma Control Questionnaire‐5; ACT, Asthma Control Test; ER, emergency room; GINA, Global Initiative for Asthma; HD, high‐dose; HTN, hypertension; ICS, inhaled corticosteroid; LABA, long acting β2 agonist; LAMA, long‐acting muscarinic antagonist; MD, medium‐dose; MPR, medication possession ratio; OCS, oral corticosteroids; QoL, quality of life; RI, reliever inhaler.

The National Review for Asthma Deaths (NRAD), carried out by the Royal College of Physicians in the UK, assessed the factors and circumstances leading to asthma‐related deaths (*n* = 195) to address these gaps to improve asthma care in the UK [18]. It was found that more than half of the people who died (57%) were not under specialist care and 10% died within one month of being discharged from the hospital and many of them were being treated for mild or moderate asthma. Notably, 21% who died had visited a hospital ER due to asthma at least once in the previous year and, of these, 58% had visited twice or more. This report also highlighted the disturbing trend of over‐prescription of SABA and under‐prescription of controller treatment and inhaled corticosteroid (ICS) [18]. Additionally, 14% were either prescribed long‐acting beta‐agonist (LABA) monotherapy or a single component LABA bronchodilator at the time of death indicating inappropriate prescription of medication. Moreover, patient perception and recognition of risk factors were observed to play an important role in asthma control [18]. Modifiable patient factors including non‐compliance to treatment, skipping physician review, and smoking were observed in 65% patients [18].

Between 2004 and 2014, there was a global surge in the diagnosis of asthma with 100 million new patients. During the same period, nearly half of the burden was reported from Southeast Asia and Western Pacific regions (46%; 45.7 and 61.2 million, respectively) [19]. One of the first surveys to assess the burden of asthma in the Asia‐Pacific region was “The Asthma Insights and Reality in Asia‐Pacific (AIRIAP)” study carried out in eight countries in early 2000 [20] (Table 1). The AIRIAP survey (*n* = 3207) reported that almost half of these patients had daytime symptoms and 44% had disturbed sleep at night due to asthma symptoms. Only around a tenth of the participants (14%) used ICS. This study underscores the mismatch in the self‐perceived and physician assessment of asthma control. About a third of respondents (34%) reportedly considered their asthma well‐controlled, however, their assessment as per the American Thoracic Society questionnaire with additional questions on activity limitation and healthcare resource utilization in the survey suggested severe persistent asthma.

Phase 2 of AIRIAP was carried out in 2006 w patients (n=4805) in Malaysia, Singapore, India, Sri Lanka, Philippines, mainland China, Hong Kong, Taiwan, Korea, Thailand, Vietnam, and Indonesia [21] (Table 1). About a third of participants (35%) had uncontrolled asthma, while 62% of participants had partly‐controlled asthma and only 3% had controlled asthma as assessed by GINA symptom control questionnaire. Patients with partly‐controlled and uncontrolled asthma had at least 4–7 fold higher hospitalization rates than patients with controlled asthma [21].

Despite advances in asthma management, studies have shown that due to its increasing prevalence and morbidity, asthma contributes significantly to the economic burden on healthcare infrastructure [22, 23, 24, 25]. The Asia‐Pacific Asthma Insights and Management (AP‐AIM) survey, (*n* = 3630 aged 12 years and above) in 2011 across nine countries in Asia‐Pacific region highlighted that 90% of patients remain uncontrolled (partly or otherwise) according to the GINA definition of asthma control [23] (Table 1). Among the participating countries, Singapore had the highest proportion of controlled patients (14%) followed by Australia (13%). Predictably, the uncontrolled and partly‐controlled patients had higher unscheduled hospital visits, greater healthcare resource utilization, and loss of productivity than the well‐controlled asthma cohort. The use of oral corticosteroids (OCS) was found to be 15‐fold higher in the partly‐controlled group than the well‐controlled patients. Moreover, partly‐controlled and uncontrolled patients reported a greater number of missed days at school/work than the well‐controlled patients. In Malaysia (*n* = 413) almost a third of these patients (30%) had uncontrolled asthma, followed by 63% being partly‐controlled as per GINA definition of asthma control. The findings from Malaysia were comparable to the proportion of partly‐controlled and uncontrolled patients in majority of the countries in the Asia‐Pacific region [23].

Asthma control remains low in Malaysia despite recent progress in asthma treatment and increasing awareness among the public. In a study in 2020 among 1280 patients in six urban public primary‐care centers in Malaysia, it was found that 63% of patients were inadequately controlled [26]. Among those with poorly controlled asthma (27%), 82% were on an inhaled controller in the previous year. Only 16% of patients had been given a written asthma action plan although a majority (92%) had received asthma education [26]. Similarly, in another study conducted at a primary care center in Malaysia among 102 patients, it was found that only 39% were controlled when evaluated according to GINA definition. Moreover, 34% had partly‐controlled, 27% had uncontrolled asthma and more than 50% of these patients had experienced exacerbations and nocturnal awakening due to asthma [27].

The Asthma Control Level in Primary Care Setting in Malaysia (ASCOPE) study across 14 primary care centers (*n* = 1011) also draws our attention to the current situation of asthma care [28] (Table 1). One‐fifth of the patients were found to be uncontrolled and 38% were partly‐controlled based on GINA symptom control tool assessment [28]. Patients with uncontrolled asthma had higher incidences of unscheduled hospital or ER visits (mean 3.9 times) and exacerbations (mean 7.6 times) highlighting the asthma unmet need at the primary care level [28].

## BARRIERS TO ACHIEVING OPTIMUM ASTHMA CONTROL

**FIGURE 1:**
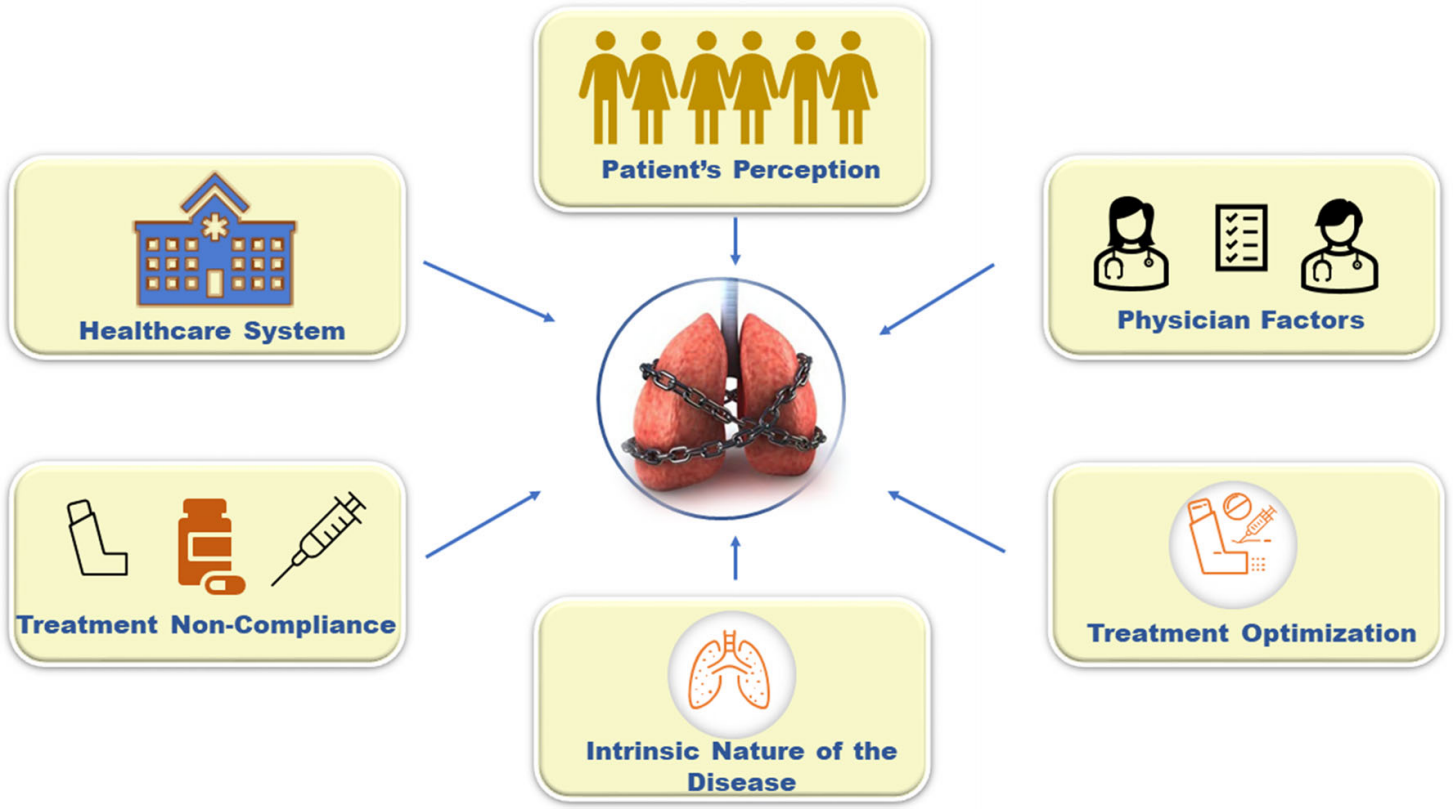
**Factors contributing to uncontrolled asthma**. Patient's perception of asthma control, treatment non‐compliance, treatment optimization, intrinsic nature of the disease, physician factors and the healthcare system are some of the noteworthy barriers in achieving optimum asthma control [19, 20, 22, 23, 27, 29, 30, 38, 39].

The challenge of uncontrolled asthma is multifaceted. The barriers to achieving optimum asthma control may be attributed to the following factors: patient's perception of asthma control, non‐compliance to treatment, physician factors and the healthcare system, treatment optimization and the intrinsic nature of the disease (Figure 1 and Table 1).

### Patient's perception of asthma control

As with any other chronic disease, patient's perception, and behavior influence self‐management of disease and its prognosis [29, 30]. The REcognise Asthma and LInk to Symptoms and Experience (REALISE) survey among 8000 patients in 11 European countries highlights patients’ attitude as a vital factor for achieving good asthma control [29] (Table 1). This survey draws our attention to the considerable gap between patient‐perceived asthma control and GINA defined control. Surprisingly, among those patients whose asthma was uncontrolled according to the GINA defined criteria, 80% of respondents considered their asthma to be well‐controlled and over two‐thirds did not consider their condition as serious. Moreover, 80% of survey participants who perceived their asthma under control had experienced acute exacerbation in the previous year and more than half (56%) reportedly had daytime activity limitations due to asthma. Most of the respondents were unable to associate symptoms with poor asthma control as around 91% of respondents perceived their asthma to be controlled whereas only 20% had their asthma under control as per GINA criteria. Almost seven in 10 patients had used their reliever inhaler at least once or more in the preceding week of this survey, and more than half of the respondents (55%) had experienced night‐time awakening due to asthma symptoms within the same duration. Notably, frequent reliever use was widespread with 4 out of 10 respondents using it three or more times in the preceding week. Interestingly, frequent reliever usage (≥10 times in the previous week) was highest for respondents on Maintenance and Reliever Therapy (MART) regime, suggesting uncontrolled asthma in this patient cohort [29]. Thus, many patients fail to associate worsening symptoms and frequent reliever use with poor asthma control.

Findings of REALISE‐ASIA survey (*n* = 2467; aged 18–50 years) were similar to its European counterpart [30] (Table 1). Interestingly, 89% of the respondents perceived their asthma to be well‐controlled, although only 19% had asthma under control as per GINA criteria [30]. Moreover, 73% of participants reported OCS use in the past 12 months, with the proportion being almost double in the uncontrolled group versus the controlled asthma cohort (88% vs. 47%). Almost 99% of uncontrolled and 69% of partly‐controlled patients reported at least one night awakening due to asthma. None of the patients with controlled asthma reported daytime activity limitations. On the contrary, half of the partly‐controlled (50%) and over 95% of uncontrolled patients had an impact on their daytime activities for at least one day due to asthma. Collectively, these disturbing figures indicate that uncontrolled and partly‐controlled asthma adversely affects the quality of life (QoL) for this group of patients. It was also observed that around 45% of respondents (*n* = 1887) who owned a reliever inhaler were using it more than twice per week. However, only about 14% of patients in the survey used their controller inhaler daily in the week before the survey [30]. Based on the patients’ perception and behavior in the REALISE Asia study, they were further stratified into five attitudinal clusters or patient types to develop a profiling tool to guide asthma management decisions for optimum outcomes: Well‐adjusted: at least partly‐controlled (Cluster 1); Rejector: in denial about symptoms (Cluster 2); Lost: Adrift and poorly‐controlled (Cluster 3); Endurer: tolerating with poor control (Cluster 4); and Worrier: worried with multiple symptoms (Cluster 5) [19]. Identifying the patient type based on the psycho‐social factors might help physicians in customizing the treatment to achieve the best patient outcomes.

A more recent survey, “Still Fighting for Breath,” echoed a similar concern albeit in patients with severe persistent asthma (*n* = 1333) and included both adults (aged > 18 years) and caregivers for children (aged 6–17 years) hailing from nine countries across continents (Canada, Brazil, Japan, Portugal, UK, Germany, France, Italy, and Spain) [31] (Table 1). A large discrepancy was observed between the proportion of patients who perceived their asthma to be well‐controlled (42%) and the proportion of patients who were found to be well‐controlled as per GINA assessment (6%) [31].

A recent study (*n* = 172) at tertiary care centers in Malaysia highlighted similar unmet needs as 68% of patients were found to be uncontrolled and 49% had a prior acute exacerbation history [32]. Despite this, 75% of patients perceived their asthma to be controlled thereby highlighting the mismatch between reality and patients’ attitude toward their symptoms [32]. Other independent studies carried out at tertiary care centers in Malaysia also highlight the importance of patient behavior and perception in achieving optimum asthma control [33, 34, 35].

### Non‐compliance to treatment

In Malaysia's ASCOPE study, treatment adherence was poor in the majority of patients, and non‐compliance more prevalent in the uncontrolled group as compared to the well‐controlled (56% vs. 44%) [28] (Table 1). Treatment adherence to prescription medication remains low in most patients with asthma even in tertiary care centers in Malaysia [32]. An important challenge was incorrect usage of inhalers in almost 20% of the patients. As expected, incorrect inhaler technique and SABA usage were found to be more prevalent in uncontrolled patient cohort [28].

In Malaysia, the Ministry of Health had taken important initiatives in 2004 to provide counselling and education to patients with chronic diseases including asthma in the form of dedicated centers called Medication Therapy Adherence Clinics (MTAC). With the overall adherence to treatment being low for chronic diseases, the objective of MTAC was to achieve an increase in treatment adherence among such patients [34, 36, 37]. In a survey in 2017 that included Malaysian Healthcare Professionals (HCPs), incorrect inhaler techniques followed by non‐compliance to treatment remained to be the biggest contributors to uncontrolled asthma [38]. Additionally, over 60% of physicians reported that more than 40% of their patients were anxious about using inhalers due to various reasons including social stigma, misbeliefs, awareness, cost etc [38].

### Physician factors and the healthcare system

Global studies have demonstrated that treating physicians tend to overrate their patients’ asthma control [39, 40]. The Global Asthma Physician Survey (GAPS) in 2017 among General Practitioners and Internal Medicine physicians (*n* = 1809) routinely seeing patients with asthma (≥4 in a month) in six developed countries to evaluate physician perception of asthma control [40] (Table 1). It was observed that asthma action plan (AAP) was seldom employed by physicians to aid patients’ asthma management and only 37% were provided with one [40]. Only 1 in 10 physicians used validated patient‐reported questionnaires (such as Asthma Control Test or ACT) to monitor asthma control, and instead relied more on monitoring selected symptoms, exacerbations, and/or lung function measurements. Awareness of single maintenance and reliever therapy (SMART) and MART varied among countries (56%–100%); although SMART/MART remained most frequently prescribed (72%) however majority (91%) co‐prescribed a SABA at least some of the time [40]. In a separate cross‐sectional survey, carried out in 29 primary and secondary care facilities in Japan, to assess the discrepancy between patient (*n* = 1697) and physician perception of asthma control, patients were allowed to fill ACQ‐5 (Asthma Control Questionnaire‐5) before a physician review [39] (Table 1). Interestingly, 52% of the patients had rated for a well‐controlled asthma on the ACQ‐5 while physicians had overestimated it at 80% [39]. One of the possible reasons for this discrepancy could have been that the patients included in this study did not experience an exacerbation in the previous 4 weeks, hence leading the treating physicians to believe that the patients have asthma under control [39]. However, this does not necessarily lower the risk of future exacerbation [1, 41]. Therefore, it is recommended to perform assessment for future exacerbations regularly using validated asthma questionnaires along with lung function tests to help physicians evaluate patients’ actual asthma control [1, 41].

Physician perception on the control of chronic diseases in their patients plays an important role in disease management. In a survey among 375 physicians with an average of 16 years of clinical experience, conducted in 2014, only 55% checked the inhaler technique of patients and almost 30% of these patients had incorrect inhaler technique [42]. In a nationwide survey in Malaysia (*n* = 226) in 2003, it was found that 98% of doctors from both public and private healthcare centers prescribed SABA (both inhaled and oral) as first line of treatment for asthma followed by ICS and LABA as second line of treatment [43]. OCS remained the third‐highest prescribed drug for asthma treatment with 54% doctors prescribing it for the third line of treatment. These prescription patterns were influenced partly by medication cost and patient stigma toward inhaler use [43]. Limitations regarding consultation time, physician motivation, patients’ receptiveness toward self‐management counselling, lack of AAP, and resources were also cited as barriers to achieving optimal asthma control in Malaysian primary care [32, 44].

### Treatment optimization

**FIGURE 2:**
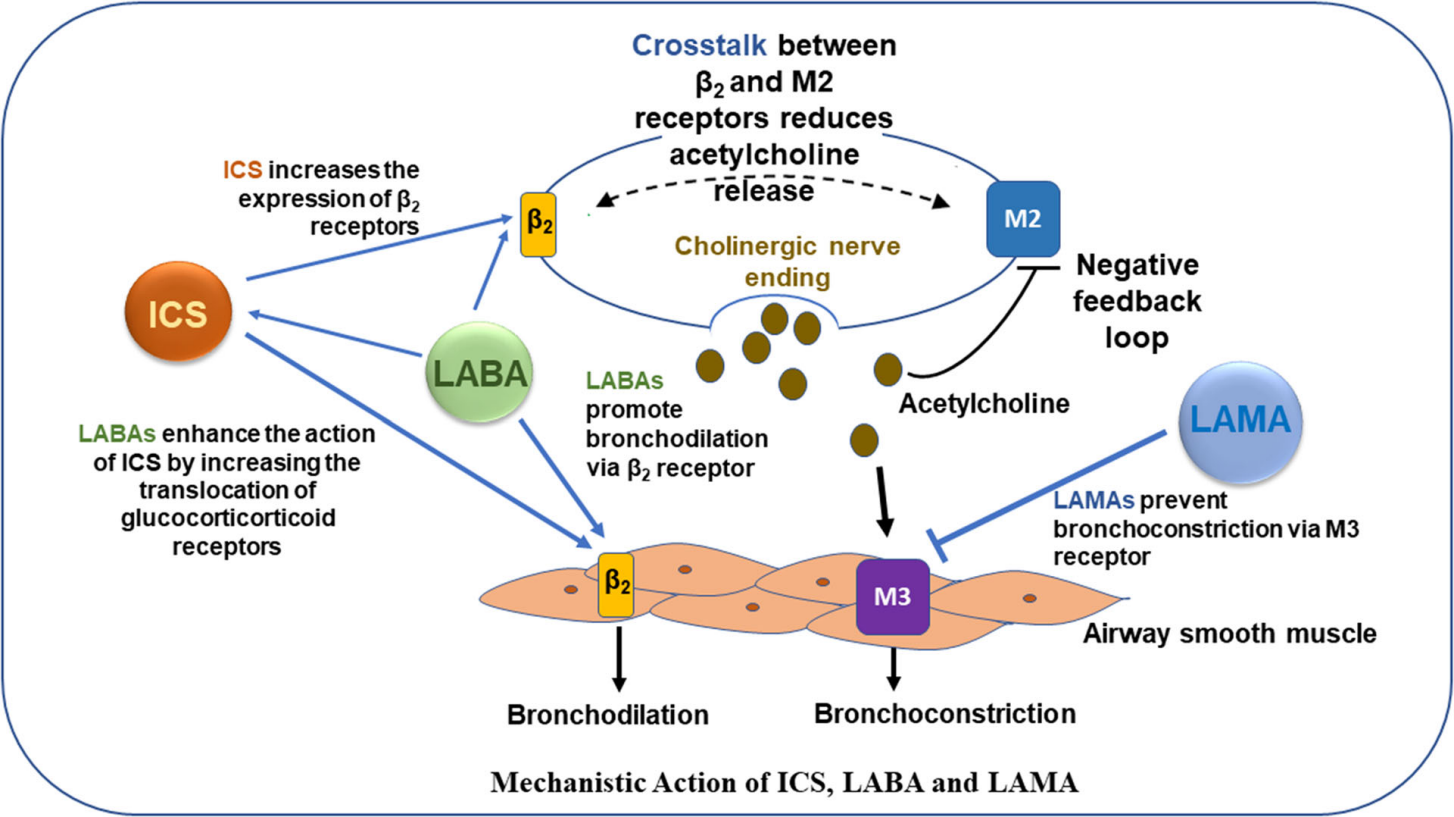
**Mechanistic action of ICS (inhaled corticosteroid), LABA (long acting β2 agonist) and LAMA (long‐acting muscarinic antagonist)**. ICS, LABA, and LAMA act synergistically to control airway inflammation and maintain normal airway tone [48, 49, 50]

GINA recommends periodic review, reassessment, and adjustment in the treatment and follow‐up of patients with asthma [1, 41]. In many instances, asthma remains uncontrolled despite treatment adherence, thus highlighting the need for timely assessment and treatment step‐up. The main inhaled therapies used in the treatment of asthma and their mechanistic action is shown in Figure 2.

According to one of the largest studies in the UK (data analyzed from Clinical Practice Research Datalink between 2006 and 2016), it was observed that at least one‐third of the patients had uncontrolled asthma [24] (Table 1). As expected, these patients were initiated either on medium (*n* = 29,229) or high dose (*n* = 16,575) ICS/LABA therapy [24]. Overall, 38% in the medium‐ and 49% in the high‐ dose ICS/LABA cohort respectively were adherent to the treatment with Medication Possession Ratio ≥ 80%. However, most of them (64% in the medium‐ and 70% in the high‐dose cohort) remained uncontrolled despite treatment adherence indicating refractory nature of asthma and/or a lack of timely treatment assessment, escalation, and introduction of additional controllers to the treatment regime. The healthcare resources were found to be used at a greater proportion by the uncontrolled asthma cohort irrespective of the ICS/LABA dose [24]. The GOAL study (*n* = 3421), a randomized, double‐blind, parallel‐group study of patients with uncontrolled asthma with treatment step‐up to achieve total and well‐controlled asthma, highlights that guideline‐derived asthma control was achieved in the majority of patients [45].

The symptomatic patients with asthma would require appropriate escalation of their maintenance treatment to achieve control and reduce exacerbation risk. Long‐acting muscarinic agents (LAMA) are shown to benefit asthma more than previously realized and can be added as a maintenance regime to ICS/LABA [46, 47, 48, 49, 50]. A review of randomized controlled trials evaluating efficacy and safety of add‐on tiotropium in children and adolescents also supports the recently expanded indication by regulatory authorities in patients 6 years or older [51]. The efficacy and safety of fixed‐dose combination (FDC) triple therapy (ICS/LABA/LAMA) for GINA Steps 4 and 5 patients has been demonstrated by the recent clinical trials [52, 53, 54, 55, 56, 57, 58, 59, 60]. Therefore, the GINA guidelines have expanded the scope of asthma treatment since 2021 to include other LAMA molecules like glycopyrronium, umeclidinium, and FDC triple therapy for patients with asthma [1, 41, 54, 61, 62]. In certain severe asthma conditions, biologics such as omalizumab (anti‐IgE), mepolizumab, reslizumab and benralizumab (anti‐IL‐5 pathways), and dupilumab (anti‐IL‐4/IL‐13) represent major advances in asthma care [63].

The cost of regular asthma treatment can be prohibitive in resource‐challenged regions in Asia‐Pacific [11, 64]. Generally, drug recommendations by GINA on stepwise asthma management according to severity and symptom control are considered cost‐effective in a recent review of 72 studies [65]. It was shown that ICS was cost‐effective when compared to leukotriene receptor antagonists (LTRA) in patients with persistent asthma. For patients who are partly controlled on ICS, the addition of LABA is also shown to be cost‐effective. Nevertheless, the issue of medicinal cost is mostly relative and should be approached in the appropriate context of local medicinal cost, patient affordability, individual healthcare system, government support, and the socio‐cultural setting.

The Asia‐Pacific region may witness more acceptance and popularity of traditional and alternative forms of asthma treatments than the Western region. In the past few years, many of the “natural” products used in traditional medicine have shown good potential and demonstrated anti‐asthma properties in in‐vitro and in‐vivo models [66]. It should be noted that alternative or traditional medicine may not necessarily be cheaper than standard asthma drugs. All these issues can complicate treatment optimization in patients with asthma.

### Intrinsic nature of the asthma

Patients with non‐eosinophilic or neutrophilic severe asthma, characterized by the absence of inflammation biomarkers like high fractional exhaled nitric oxide (FeNO) and eosinophils, may often be unresponsive to steroid treatment, thereby making asthma control difficult in such sub‐type of patients [67, 68, 69, 70]. These patients have asthma that is not associated with allergy or falls under non‐type‐2 inflammation. The exact etiology of neutrophilic asthma is not well‐understood. It has been shown in an independent study (*n* = 1197) that neutrophilic airway inflammation may be related to persistent airflow limitation [71]. Sputum neutrophilia was associated with lower pre‐ and postbronchodilator FEV1 (Forced expiratory volume in the first second) indicating a decline in lung function [71]. Moreover, it was shown that a 10‐fold increase in neutrophil count was associated with a 92 ml reduction in postbronchodilator FEV1 [71]. Unlike type‐2 asthma, the refractory nature of neutrophil‐driven asthma poses a considerable clinical challenge and one without effective treatment alternatives. GINA recommends the use of oral azithromycin, only after specialist referral, as an add‐on to high dose ICS/LABA for severe persistent asthma and this may result in fewer exacerbations and improved QoL [1, 41, 70, 72].

## OVERCOMING BARRIERS AND FUTURE DIRECTIONS

**FIGURE 3:**
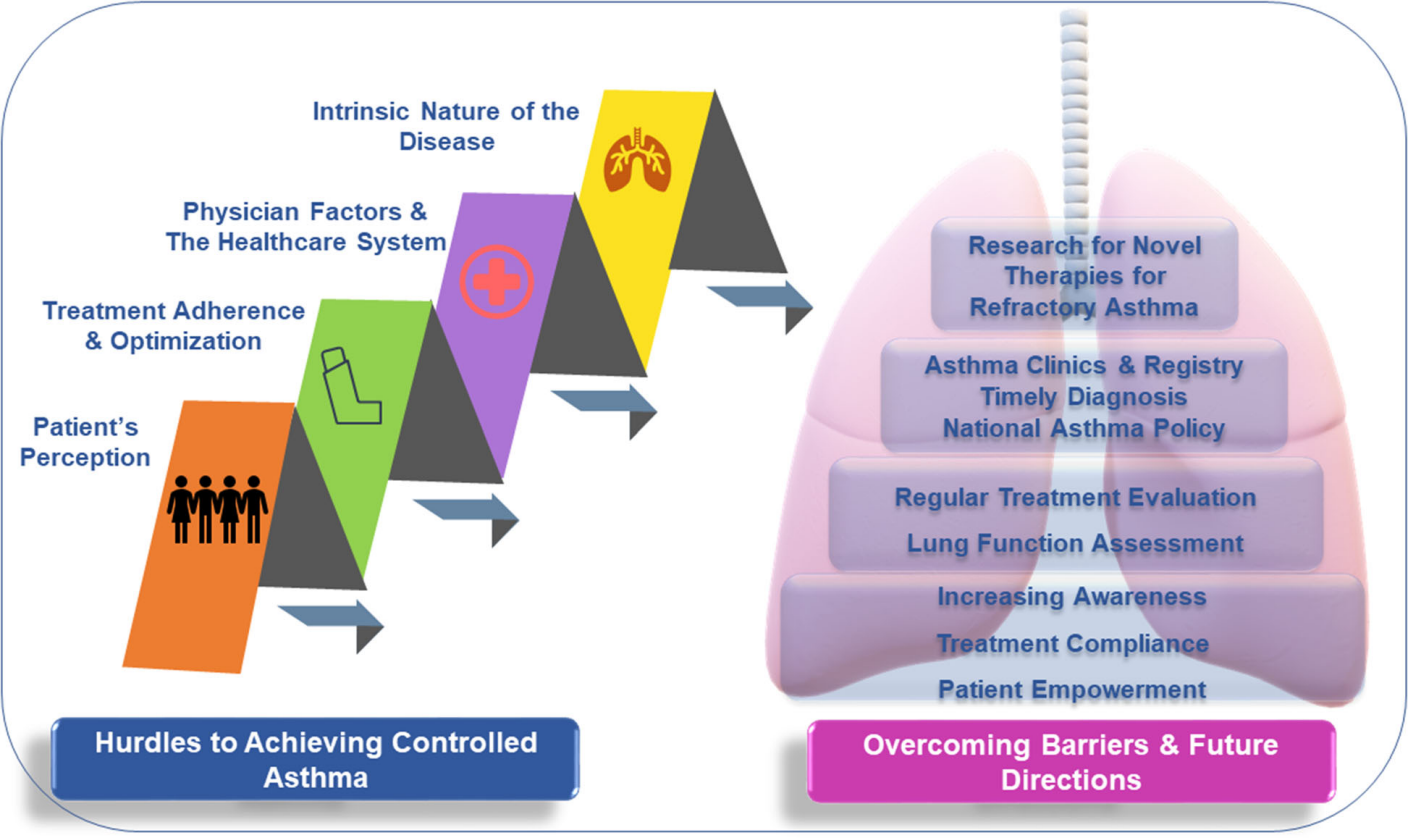
**Barriers to achieving asthma control and suggested ways of overcoming these barriers**. Challenges to achieving optimum asthma control include patient factors (behaviour, perception and treatment non‐compliance), physician factors, and the healthcare infrastructure. An integrated approach of elevating awareness about asthma management among patients and healthcare professionals, improving healthcare infrastructure along with national asthma policies might address some unmet needs

The impact of uncontrolled asthma is manifold both for the individual and the society. Treatment compliance, regular follow‐ups especially within a few days of hospital discharge, objective assessment of asthma by validated questionnaires and regular lung function assessments have shown to improve asthma control. To achieve this, concerted efforts are required to increase awareness and motivation among all stakeholders as shown in Figure 3. Patient factors including treatment compliance, attending regular physician reviews and perception of asthma in general can be addressed by increasing awareness about asthma and educating patients as well as their families. The advent of triple therapy may be helpful in reducing the inhaler burden of patients, which often can come in the way of treatment adherence and simplify the medication posology. Shared short‐term goals between patient and treating physician can help in improving treatment compliance and motivating the individual. Providing a personalized written asthma action plan is important in empowering the patients to better manage their asthma. An asthma action plan with information about current medications, triggers, signs of worsening symptoms, and how to seek help during emergencies may enable patients and their caregivers to effectively self‐manage the condition. Professional counselling services should be made available, where possible, for patients with asthma to help them overcome their fears, myths, and social stigma associated with this chronic condition. Physicians should be encouraged to use guideline recommended asthma questionnaires at each patient visit and perform spirometry periodically to determine asthma control and guide treatment decision.

Patients requiring frequent nebulization and OCS should be referred promptly for a structured specialist review. Patients’ inhaler technique evaluation at each follow‐up visit is paramount. Adequate treatment of comorbidities, like allergic rhinitis, nasal polyposis, etc., also plays a vital role in achieving optimum asthma control. Hence, the healthcare resources can be streamlined by setting up dedicated multidisciplinary asthma clinics where all relevant specialties including pulmonologists, ear nose throat specialists, allergists, and counsellors are available for consultation. Involving pharmacists in long‐term asthma management can help monitor reliever inhaler refills and early identification of red flags. Maintaining a consolidated electronic prescription database and asthma registry can help track patients’ medication and inhaler usage along with exacerbation history to provide suitable and timely medical intervention. In refractory asthma cases, prompt evaluation and treatment intensification with add‐on controllers and biologics might be helpful in achieving treatment goals. In resource‐challenged countries in the Asia‐Pacific region, national asthma strategies may aid in improving treatment affordability and equitable access to healthcare services.

## CONCLUSION

Asthma is one of the significant non‐communicable diseases. While strides have been made in the asthma treatment landscape, the burden of asthma continues to grow as majority of the unmet needs are not adequately addressed. Although, asthma prevalence is higher in North America and Europe, majority asthma related deaths have been reported from the Asia‐Pacific region. The unmet needs in achieving good control and preventing asthma exacerbations remain a major challenge in the Asia‐Pacific region. In addition to treatment non‐compliance and inequitable access to treatment, there are discrepancies in the patient's and physician's perception of asthma control. Concerted efforts on patient education, timely diagnosis and effective management, treatment accessibility, treatment adherence, and improving healthcare infrastructure are required to minimize morbidity and mortality related to uncontrolled asthma in the Asia‐Pacific region.

## CONFLICTS OF INTEREST

The authors declare no competing financial or non‐financial interests.

## ETHICS APPROVAL

As this is a perspective article, no ethics approval was required.

## AUTHOR CONTRIBUTIONS

Surbhi Wadhawan, Umadevi A. Muthukumaru, and Li Cher Loh were equally responsible for study conception. Surbhi Wadhawan, Umadevi A. Muthukumaru, and Li Cher Loh contributed equally to the preparation of the first and subsequent drafts of the manuscript. All authors read and approved the final version of the manuscript.

## Data Availability

No primary data was used in preparing this manuscript.
